# Effects of Medicaid Coverage on Work: Evidence From Extending Postpartum Medicaid Coverage

**DOI:** 10.1111/1475-6773.70055

**Published:** 2025-10-09

**Authors:** Ufuoma Ejughemre, Wei Lyu, George L. Wehby

**Affiliations:** ^1^ Department of Health Management and Policy University of Iowa Iowa City Iowa USA; ^2^ Department of Health Services Administration University of Alabama at Birmingham Birmingham Alabama USA; ^3^ Department of Economics University of Iowa Iowa City Iowa USA; ^4^ National Bureau of Economic Research Cambridge Massachusetts USA

## Abstract

**Objective:**

To evaluate the effects of the Family First Coronavirus Response Act (FFCRA) on work outcomes of women for whom the FFCRA effectively expanded income eligibility for Medicaid beyond 60 days postpartum by prohibiting states from redetermining Medicaid eligibility between March 2020 and March 2023.

**Study Setting and Design:**

We use a difference‐in‐differences design that leverages the differences in income eligibility between pregnancy and non‐pregnancy across states, and compares outcome changes pre–post FFCRA over these differences.

**Data Sources and Analytic Sample:**

Data come from the 2016–2022 American Community Survey. The sample includes 205,104 women aged 19–49 years who reported giving birth within the past 12 months in 41 states and Washington D.C.

**Principal Findings:**

On average, the FFCRA increased postpartum Medicaid coverage by 2.8 percentage points (95% CI: 0.7–4.8) or by 9.3% relative to the 2019 Medicaid coverage rate. In contrast, the FFCRA effects on work outcomes were small and not significant: the average effect was 0.10 percentage points for labor force participation (95% CI: −1.0 to 1.2), 0.7 percentage points for employment (95% CI: −0.02 to 1.4), 0.04 h for weekly work hours (95% CI: −0.4 to 0.5), and 0.2 percentage points for full‐time employment (95% CI: −1.1 to 1.5). These confidence intervals rule out an employment decline above 0.02 percentage points and full‐time employment decline above 1.1 percentage points. The increase in Medicaid coverage is concentrated among states with a larger difference between pregnancy and non‐pregnancy eligibility (+5.9 percentage points; 95% CI: 0.9 to 10.9) and estimates in this group also rule out relatively small declines in work outcomes.

**Conclusion:**

There is no evidence of declines in work outcomes following the increase in Medicaid coverage beyond 60 days postpartum that resulted from the FFCRA. The findings suggest that subsequent postpartum Medicaid coverage extensions for 12 months under the American Rescue Plan are unlikely to disincentivize work among beneficiaries.

**Summary**

- What is known on this topic?
  ○ Several states and the federal government have added work requirements to maintain eligibility of adult beneficiaries for Medicaid due to concerns about disincentives to work.
  ○ Work requirements have not applied to pregnancy or postpartum Medicaid eligibility or parents of young children, but whether Medicaid affects work for those groups is not well known.
  ○ The extant literature examining a range of Medicaid eligibility changes offers mixed results with some studies finding no effects on work while others reporting some dis‐employment effects.
- What this study adds?
  ○ This study examines the effects of a recent expansion in income eligibility for Medicaid beyond 60 days postpartum resulting from the Family First Coronavirus Response Act (FFCRA) on work outcomes.
  ○ Using nationally representative data and a difference‐in‐differences design, the study finds an increase in Medicaid coverage but generally no evidence of effects on work outcomes.
  ○ Effect estimates can rule out relatively small declines in employment and other work outcomes in this population following increased Medicaid coverage from the FFCRA.

## 1 Introduction

Expanding Medicaid eligibility for low‐income adults has often raised debates about potential unintended effects to discourage employment among those gaining coverage. Following the Medicaid expansions under the Affordable Care Act (ACA), 15 of 41 states that have expanded Medicaid eligibility for adults have added or are in the process of adding work requirements to maintain eligibility because of concerns about such effects [1]. Recently, the One Big Beautiful Bill Act of 2025 required work or community service for adult beneficiaries enrolled under the ACA Medicaid expansions with exemptions for parents of children 13 years or younger, pregnancy and postpartum enrollment, and some other groups. The policy concern with adding work requirements is that Medicaid eligibility may disincentivize work by decoupling health coverage benefits from employment. Conceptually, this is plausible for individuals seeking employment primarily for health coverage rather than for wages, especially if income loss is offset by other benefits such as nutritional, housing, or cash assistance from safety‐net programs.

Although conceptually plausible, the empirical evidence on whether Medicaid eligibility expansions impact work outcomes remains mixed. Several studies of the ACA Medicaid expansions have found no significant effects on work outcomes, including employment [2, 3, 4, 5], job switching [2], or full‐versus part‐time employment status [2], and hours worked [4]. However, a study employing border county comparisons reported temporary declines in employment [6]. Some studies of other Medicaid expansions or changes in eligibility suggest some disemployment effects from increased eligibility. One study of disenrolling nearly 170,000 adults from Tennessee's Medicaid program in 2005 following a process change in determining eligibility reported a large increase in employment [7]. Also, a study of Medicaid eligibility expansion in Wisconsin in 2019 for childless adults with incomes below 200% FPL reported a decline in employment among enrollees compared to those placed on a waitlist following the sudden suspension of enrollment in the program [8]. An earlier study examining the effects of Medicaid expansions for pregnant women during the late 1980s and early 1990s found declines in labor force participation, employment status, and weeks and hours worked during the year of giving birth [9].

In this paper, we examine the effects of a more recent change in determining Medicaid eligibility under the Family First Coronavirus Response Act (FFCRA) implemented during the COVID‐19 pandemic. The FFCRA prohibited states from disenrolling Medicaid beneficiaries and redetermining their eligibility between March 2020 and March 2023 in order to reduce insurance interruptions for low‐income individuals during the pandemic [10, 11]. We focus on how the FFCRA affected postpartum Medicaid coverage for women who gave birth during this period. Even though the FFCRA did not technically change income eligibility, it virtually expanded income eligibility by extending postpartum coverage beyond 60 days. Because Medicaid covers pregnant women at higher income levels during pregnancy and up to 60 days postpartum than before pregnancy or after 60 days postpartum (in all states but one), women who would have lost postpartum Medicaid coverage after 60 days due to their income exceeding non‐pregnancy eligibility [138% of the federal poverty line (FPL) in states that expanded Medicaid under the ACA or lower in states that did not expand under the ACA] remained eligible. For this specific group, the FFCRA represented a benefit akin to income eligibility expansion for coverage post 60 days.

Emerging evidence shows that Medicaid coverage rates increased beyond 60 days postpartum following the FFCRA [12, 13, 14]. However, no study has examined the effects of this coverage expansion on work outcomes. Such evidence, albeit specific to women during postpartum, is important not only because of the mixed evidence from studying prior Medicaid expansions and the continued need to understand the effects of recent and different Medicaid eligibility changes on work outcomes, but also because of the relatively large income eligibility expansion for postpartum coverage beyond 60 days under this provision. Indeed, the FFCRA extended postpartum coverage beyond 60 days by as much as 144% to 380% of the FPL depending on the state; only one state (Louisiana) covered pregnancy at the same level as non‐pregnancy adult coverage [15]. Therefore, this study contributes to the broader literature on various types of Medicaid eligibility and enrollment expansions on work. Moreover, following the unwinding of the FFCRA in March of 2023, almost all states (49 states and DC as of September 15, 2025) have continued to extend postpartum coverage from 60 days to 12 months under the American Rescue Plan (ARP) of 2021, with most of these states extending coverage up to the same income level as during pregnancy and the first 60 days [16, 17, 18]. Therefore, examining the effects of postpartum Medicaid coverage gain beyond 60 days under the FFCRA also provides early and timely evidence on the potential effects of the coverage extensions up to 12 months under the ARP on work.

This study examines the effects of gaining Medicaid coverage beyond 60 days postpartum due to the FFCRA on work outcomes. We employ data from the American Community Survey (ACS) with information on whether a woman gave birth during the past 12 months to identify our study sample. We implement a difference‐in‐differences design that leverages the variation between states in their income eligibility levels for Medicaid coverage during pregnancy (and 60 days postpartum) and non‐pregnancy. We first demonstrate the Medicaid coverage gain under this provision in this sample and then examine a range of work outcomes.

## 2 Study Data and Methods

### 2.1 Data and Sample

The data comes from the 2016–2022 ACS, which is a repeated cross‐sectional survey with a random 1% sample of the United States population [19]. The ACS collects detailed information on sampled individuals' demographic and socioeconomic characteristics, current health insurance coverage status, and work outcomes.

The analytical sample includes individuals aged 19 to 49 years who have reported giving birth within the past 12 months in response to the following question: “Regardless of your marital status, did you give birth to any child in the past 12 months?” [20]. Therefore, our sample includes women at any time in the postpartum period up to 12 months from the date of giving birth. One issue with this question is that some of the women surveyed in 2020 may have given birth in 2019 and therefore would not have been affected by the FFCRA. To address this possibility of partial effects, we conduct alternate analyses that exclude the 2020 data or estimate year‐by‐year effects as described below in detail. In addition, unlike several previous Medicaid evaluation studies that often limit the sample to low‐income individuals [2, 3, 4, 5], we do not limit the main sample to any income level because the FFCRA allowed individuals to maintain coverage even if their income exceeded Medicaid eligibility thresholds subsequent to their enrollment. Income is also endogenous to any potential changes in employment. Indeed, as we show below, Medicaid coverage rates in this sample increased across income levels during the FFCRA years despite the increase being concentrated in 51%–400% FPL range. Therefore, we consider two alternate samples, the first includes incomes in this range of 51%–400% FPL, and the second includes individuals of high school or some college education, the group with the highest Medicaid coverage gains when stratifying by education. We exclude from the sample 9 states (Idaho, Louisiana, Maine, Missouri, Montana, Nebraska, Oklahoma, Utah and Virginia) that expanded Medicaid eligibility under the ACA between 2016 and 2022 to avoid other changes in state income eligibility outside of pregnancy during the study period. The main analytical sample includes 205,104 women.

### 2.2 Outcomes

The main insurance outcome is Medicaid coverage at the survey time. Additional insurance outcomes include private coverage by type (independently purchased or employer‐sponsored) and any coverage to understand how much of the increased Medicaid coverage is from being uninsured versus crowd‐out from private coverage. We evaluate four work outcomes, all based on status at the survey time. These include a binary (0/1) indicator for labor force participation which includes employment or actively seeking employment, another indicator for being employed, the number of usual hours worked per week (including 0 h for unemployed), and an indicator for working full‐time (30 or more hours per week) versus part‐time (29 h or less).

### 2.3 Research Design and Statistical Analysis

To identify the effects of expanding Medicaid income eligibility beyond 60 days postpartum under the FFCRA, our research design leverages differences across states in Medicaid income eligibility between pregnancy and non‐pregnancy (i.e., parent eligibility level) in 2019. Since the FFCRA continued Medicaid eligibility up to the pregnancy income eligibility level beyond 60 days throughout the period between March 2020 and March 2023, more postpartum women would have benefited from this continued coverage in states that had a larger difference in income eligibility between pregnancy and non‐pregnancy. A larger value of this difference implies that the FFCRA would have affected a larger proportion of postpartum women in the state. As shown in Table A1, there is extensive variation between included states in the income eligibility difference between pregnancy and non‐pregnancy, which ranges from 23% to 242% of the FPL.

To implement this design, we estimate the following difference‐in‐differences model:

(1) $$Y_{\mathrm{ist}}=\alpha +\beta \left({\text{ΔElig}}_{s}\times {\text{Post}}_{t}\right)+X_{\mathrm{ist}}\Gamma +\theta_{s}+\omega_{t}+\epsilon_{\mathrm{ist}}$$

$Y_{\mathrm{ist}}$ is one of the study outcomes for individual i in state s interviewed in year t. ${\text{ΔElig}}_{s}$ is a continuous state‐level measure of the difference in Medicaid income eligibility between pregnancy and out of pregnancy (i.e., parent eligibility level) in 2019 shown in Table A1. A larger value of $\text{ΔEli}g_{s}$ implies that the FFCRA would have affected postpartum Medicaid coverage (beyond 60 days) for a larger proportion of women in that state. ${\text{Post}}_{t}$ is equal to 1 for years 2020 through 2022 (years when FFCRA was effective), and 0 for 2016–2019. In an alternate specification, ${\text{Post}}_{t}$ is equal to 1 for years 2021–2022 and 2020 data are dropped from the model due to potential partial effects in 2020 based on the question of giving birth within the past 12 months as noted above. $X_{\mathrm{ist}}$ includes a set of conceptually relevant individual characteristics that may affect outcomes including maternal age (added as 0/1 indicators for each year), education (less than high school, high school, some college, college and above), race/ethnicity (non‐Hispanic White, non‐Hispanic Blacks, non‐Hispanic other races, Hispanics), marital status (married or not), number of adults in the household (binary indicators with a cap of 5 or more adults), number of children in household (binary indicators with a cap of 5 or more children), and citizenship status. In a sensitivity check, we add the state‐level unemployment rate (obtained from the Bureau of Labor Statistics) as a covariate to capture differences between states in labor market trends during this period [21]. $\theta_{s}$ includes state fixed effects (0/1 indicators) that account for time‐invariant differences across states. $\omega_{t}$ includes year fixed effects which capture national trends shared across states. The coefficient (β) of the interaction between $\text{ΔEli}g_{s}$ and $\mathrm{Pos}t_{t}$ is the intention‐to‐treat (ITT) effect of the Medicaid coverage expansion effect from the FFCRA on the study outcomes. Because the effect is proportional to $\text{ΔEli}g_{s}$ and to make the results more interpretable, we estimate the average treatment effect as $\beta \times \bar{\text{ΔEli}g_{s}}$, where $\bar{\text{ΔEli}g_{s}}$ is the mean difference between pregnancy and non‐pregnancy Medicaid income eligibility across included states which is 97% of the FPL. We also provide estimates scaled by the 75th and 90th percentiles of the state differences between pregnancy and non‐pregnancy eligibility.

Additionally, we estimate the following event‐study difference‐in‐differences model to examine effect changes over time and assess outcome pre‐trends:

(2) $$Y_{\mathrm{ist}}=\alpha +\sum_{t\neq 2019}\delta_{t}\left(\Delta {\text{Elig}}_{s}\times {\text{Year}}_{t}\right)+X_{\mathrm{ist}}\Gamma +\theta_{s}+\omega_{t}+\epsilon_{\mathrm{ist}}$$

The event study adds interactions between $\Delta {\text{Elig}}_{s}$ and 0/1 indicators for all years except for 2019 as the reference year. $\delta_{2020}$, $\delta_{2021}$, and $\delta_{2022}$ estimate the policy effects separately in 2020, 2021, and 2022, respectively. $\delta_{2016}$, $\delta_{2017}$, and $\delta_{2018}$ capture differences in pre‐trends by treatment intensity (in 2016, 2017, and 2018, respectively, relative to 2019) to evaluate the difference‐in‐differences design validity (i.e., the parallel trends assumption). Because it estimates year‐specific effects, the event‐study specification allows for the FFCRA effect to differ in each implementation year, also addressing the issue of some respondents in 2020 giving birth in 2019 and therefore not affected by the FFCRA.

We further evaluate potential effect heterogeneity by the difference between pregnancy and non‐pregnancy eligibility that re‐estimates model (1) separately for states above the median of that difference and those at or below that median (state group assignment shown in Table A1). Moreover, because of differences in income and pregnancy Medicaid take‐up by age, marital status, and race/ethnicity, we also evaluate potential heterogeneity in effects across these demographic factors. Regressions are estimated with linear least squares weighted by the ACS sampling probability weights, and standard errors are clustered at the state level. Estimates are considered statistically significant at 5%.

## 3 Results

### 3.1 Descriptive Analysis

We begin by showing in Table 1 the sample postpartum Medicaid coverage rates by income, education, and year and the difference between 2022 (the last study year) and 2019 (the year before FFCRA). Medicaid rates increased noticeably after FFCRA implementation, especially in 2021 and 2022, with the largest increase by 4% to 6% between 2019 and 2022 among women with incomes between 51% and 400% FPL. Women from other income groups had a much lower increase generally by around 1%. By education, the largest increase in Medicaid rates was for those with high school or some college education (at 4% to 6%) with apparent decline for those with less than high school and an increase for college graduates by about 2%. The lack of an apparent increase in postpartum Medicaid coverage between 2019 and 2022 for women with incomes up to 50% FPL and less than a high school education is likely due to this policy affecting primarily the continued eligibility for Medicaid for women at income levels who otherwise would have been ineligible after 60 days as noted above.

**TABLE 1:** Descriptive statistics showing postpartum women's medicaid rates (%) by income by year for full sample.

	2016	2017	2018	2019	2020	2021	2022	Difference between 2022 and 2019
Income as % FPL								
0–50	67.60	68.66	65.30	69.46	67.37	68.45	70.49	1.03
51–100	62.55	62.79	60.66	61.59	63.17	66.60	65.70	4.11
101–200	45.69	45.88	44.70	44.63	47.86	52.33	50.95	6.32
201–300	26.17	26.65	26.18	27.55	28.04	33.11	32.62	5.07
301–400	14.56	15.23	14.05	13.39	17.18	17.78	19.85	6.46
401–500	9.22	8.73	9.42	10.44	10.46	11.44	11.63	1.19
501–600	7.01	6.09	7.26	6.83	8.25	9.55	8.47	1.64
601–700	5.37	5.11	6.11	5.14	6.23	6.05	6.15	1.01
701–800	5.05	4.16	3.68	4.35	4.25	3.85	4.46	0.11
801–900	2.40	5.30	4.45	4.48	3.11	7.01	3.94	−0.54
901–1000	1.76	2.20	4.15	2.87	4.31	5.05	4.77	1.90
1001 and above	4.41	3.94	2.76	2.03	2.41	4.72	3.67	1.64
Education								
Less than high school	53.24	51.31	50.50	50.87	49.68	49.06	47.23	−3.64
High school	48.57	47.08	45.97	45.42	47.28	51.32	49.64	4.22
Some college	34.92	35.32	33.39	32.71	33.63	37.94	38.76	6.05
College and above	8.66	8.40	7.88	7.58	8.96	10.64	10.52	2.94

*Note:* The descriptive statistics are derived from the analytical sample among women aged 19 to 49 years old who gave birth within the past 12 months. All statistics are weighted using the sampling weights.

Abbreviation: FPL, Federal Poverty Level.

Table A2 reports the sample descriptive statistics. In 2020–2022, about 30% had Medicaid coverage, 62% had private coverage (2% had both private and Medicaid coverage), and 8% were uninsured. Nearly 62% were in the labor force, 65% were employed, and 40% had full‐time jobs; the average number of hours worked per week was 22 h (including non‐workers).

### 3.2 FFCRA Effects on Postpartum Medicaid Coverage and Work Outcomes

#### 3.2.1 Main Sample

Table 2 shows the difference‐in‐differences estimates of the FFCRA effects on postpartum Medicaid coverage and work outcomes in the main sample with effects scaled by the mean difference between pregnancy and non‐pregnancy income eligibility across states. There is an increase in postpartum Medicaid coverage rate during 2020–2022 versus 2016–2019; the average increase is 2.8 percentage‐points (95% CI: 0.7 to 4.8). The increase in Medicaid coverage is slightly more pronounced (+3.1 percentage points; 95 CI: 1.0 to 5.2) when excluding year 2020 (Table A3) as expected, considering that some respondents in 2020 would have given birth in 2019 and would not have been affected by the FFCRA. The increase in Medicaid coverage appears to be largely from the group that would have been uninsured rather than crowd‐out from private coverage since there is a close increase in any coverage rate and small and statistically insignificant changes in private coverage (Table A4 including 2020 data and Table A5 excluding 2020 data). This increase in Medicaid coverage is meaningful, representing a 9.3% increase relative to the 2019 rate. The Medicaid coverage increase is more pronounced in states with a larger difference in income eligibility between pregnancy and non‐pregnancy, including by 3.7 and 5 percentage‐points at the 75th and 90th percentiles of this difference, respectively (Table A6).

**TABLE 2:** Effects of FFCRA medicaid continuous coverage provision on medicaid coverage and work outcomes, women aged 19–49 years with a child born within the past 12 months for the full sample, 2016–2022 ACS.

	Medicaid coverage	Labor force participation	Employment status	Working hours per week^a^	Fulltime employment status^a^
Effect estimate (2020–2022)	2.8**	0.10	0.7	0.04	0.2
SE	(1.0)	(0.5)	(0.4)	(0.2)	(0.6)
95% CI	(0.7, 4.8)	(−1.0, 1.2)	(−0.02, 1.4)	(−0.4, 0.5)	(−1.1, 1.5)
*N*	205,104	205,104	205,104	205,104	205,104
Outcome Mean (2016–2019)	29.7	65.2	60.6	25.1	55.5

*Note:* This table reports the effects of the FFCRA Medicaid continuous coverage provision on postpartum health insurance and work outcomes. Estimates are from a difference‐in‐differences regression leveraging differences across states in income eligibility between pregnancy and non‐pregnancy and over time (2020–2022 versus 2016–2019). Effects are scaled to the average difference in this income eligibility in 2019 (97% FPL). The model adjusts for maternal age, education, race/ethnicity, marital status, number of adults and children in the household, unemployment rates, US citizenship status, state, and year fixed effects. All regressions are estimated using the least squares with the sampling probability weights provided in the ACS data.

Abbreviations: ACS, American Community Survey; CI, Confidence Interval; FFCRA, Families First Coronavirus Response Act (FFCRA); FPL, Federal Poverty Level; SE, Standard Errors.

^a^ Outcomes include zeros for unemployed individuals and those not in the labor force.

** *p* < 0.01.

The FFCRA effect estimates for work outcomes are small and not statistically significant. Importantly, the effect estimates are precise enough to rule out relatively small declines in work outcomes associated with the FFCRA. Specifically, the 95% confidence intervals rule out declines of more than 1.0 percentage‐point in labor force participation, 0.02 percentage‐points in employment, 0.4 work hours per week, and 1.1 percentage‐points in full‐time employment status when scaling the effect by the mean difference between pregnancy and non‐pregnancy income eligibility (Table 2). Relative to the 2019 outcomes, these declines represent about 1.5% of labor force participation rate, 0.03% of employment rate, 1.6% of weekly work hours, and 2.0% of full‐time employment rate. Considering the 9.3% increase in Medicaid coverage on average, these are relatively small declines that can be ruled out based on the estimate precision and study power. Similarly, small declines can be ruled out when scaling the effect by a larger difference between pregnancy and non‐pregnancy eligibility, including declines of more than 1.7 percentage‐points in labor force participation, 0.7 percentage‐points in employment, 0.7 work hours per week, and 2.0 percentage‐points in full‐time employment (Table A6).

**FIGURE 1:**
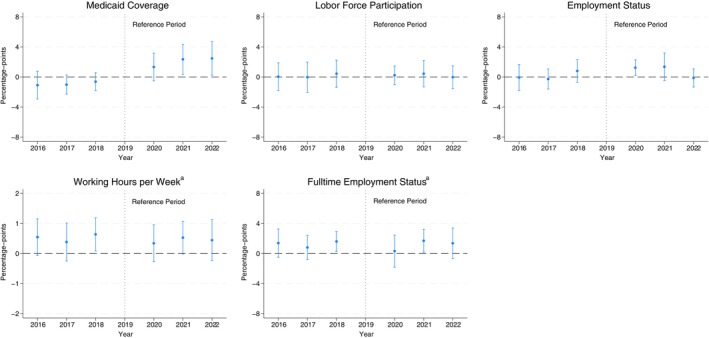
Year‐by‐year effects of FFCRA medicaid continuous coverage provision on medicaid coverage and work outcomes, women aged 19–49 years with a child born within the past 12 months for the full sample, 2016–2022 ACS. This graph shows the year‐by‐year effects (dots) and the 95% confidence intervals (bars) of FFCRA Medicaid continuous coverage provision on postpartum insurance outcomes. Estimates are from an event study difference‐in‐differences regression leveraging differences across states in income eligibility between pregnancy and non‐pregnancy and over time (2020–2022 vs. 2016–2019). Effects are scaled to the average difference in this income eligibility in 2019 (97% FPL). All models control for maternal age, education, race/ethnicity, marital status, number of adults in household, number of children in household, US citizenship status, state and year fixed effects. All regressions are estimated using the least weighted squares with the sampling probability weights provided in the ACS data. ^a^Outcomes include zeros for unemployed individuals and those not in the labor force. ACS, American Community Survey; CI, Confidence Interval; FFCRA, Families First Coronavirus Response Act (FFCRA); FPL, Federal Poverty Level; SE, Standard Errors.

Figure 1 shows the event‐study estimates of the year‐by‐year FFCRA effects on postpartum Medicaid coverage and work outcomes relative to 2019 (scaling by the mean difference between pregnancy and non‐pregnancy eligibility). Consistent with the descriptive analysis (Table 1), the increase in Medicaid coverage is concentrated in 2021 and 2022 relative to 2019, on average by 2.4 (95% CI: 0.4 to 4.3) and 2.5 points (95% CI: 0.24 to 4.7) percentage‐points, respectively. There are no apparent corresponding effects on most work outcomes from this Medicaid coverage increase, similar to the estimates that pool the post‐FFCRA years. Estimates for these outcomes are not statistically significant but also precise enough to rule out small to moderate effects on work outcomes. Overall, there are no apparent pre‐trends that would indicate any systematic bias in the FFCRA effects on these outcomes; the estimates during pre‐FFCRA years are all small and statistically insignificant.

#### 3.2.2 Alternate Samples

Table 3 shows the difference‐in‐differences estimates for the alternate samples, which are incomes within 51%–400% FPL, and high school or some college education These estimates are consistent with the main sample results. These samples show an increase in Medicaid coverage rates with a more pronounced increase by nearly 4 percentage‐points (95% CI: 1.2 to 6.7) among women with high school or some college education. For work outcomes, estimates are generally small and not statistically significant in these alternate samples except for a small increase in employment by 1.3 percentage‐points among those with 51%–400% FPL income. Similar to the main sample, effect estimates are precise enough to rule out relatively small declines in work outcomes.

**TABLE 3:** Effects of FFCRA medicaid continuous coverage provision on medicaid coverage and work outcomes, women aged 19–49 years with a child born within the past 12 months for the samples 51%–400% FPL and high school or some college education, 2016–2022 ACS.

	Medicaid coverage	Labor force participation	Employment status	Working hours per week^a^	Fulltime employment status^a^
Panel A: Income 51%–400% FPL					
Effect estimate (2020–2022)	2.6*	0.6	1.3**	0.3	1.0
SE	(1.2)	(0.6)	(0.5)	(0.2)	(0.7)
95% CI	(0.2, 5.0)	(−0.7, 1.9)	(0.3, 2.2)	(−0.1, 0.8)	(−0.3, 2.3)
*N*	117,693	117,693	117,693	117,693	117,693
Outcome mean (2016–2019)	36.2	60.6	55.8	22.9	50.4
Panel B: High school or some college education					
Effect estimate (2020–2022)	4.0**	0.3	1.2	0.2	0.9
SE	(1.4)	(0.9)	(0.6)	(0.3)	(0.8)
95% CI	(1.2, 6.7)	(−1.6, 2.1)	(−0.10, 2.5)	(−0.4, 0.9)	(−0.7, 2.5)
*N*	102,288	102,288	102,288	102,288	102,288
Outcome mean (2016–2019)	39.6	62.6	56.8	23.4	51.7

*Note:* This table reports the effects of FFCRA Medicaid continuous coverage provision on postpartum health insurance and work outcomes. Estimates are from a difference‐in‐differences regression leveraging differences across states in income eligibility between pregnancy and non‐pregnancy and over time (2020–2022 vs. 2016–2019). Effects are scaled to the average difference in this income eligibility in 2019 (97% FPL). The model adjusts for maternal age, education, race/ethnicity, marital status, number of adults in the household, number of children in the household, US citizenship status, state and year fixed effects. All regressions are estimated using the least squares with the sampling probability weights provided in the ACS data.

Abbreviations: ACS, American Community Survey; CI, Confidence Interval; FFCRA, Families First Coronavirus Response Act (FFCRA); FPL, Federal Poverty Level; SE, Standard Errors.

* *p* < 0.05.

** *p* < 0.01.

*** *p* < 0.001.

^a^ Outcomes include zeros for unemployed individuals and those not in the labor force.

### 3.3 Additional Analyses

Table A7 reports the results of an alternate model that adds the state unemployment rate as a covariate to capture state‐specific labor market trends. The results are similar to the main estimates. Table A8 shows the estimates separately for states above the median difference between pregnancy and non‐pregnancy income eligibility and those at or below the median. The increase in Medicaid coverage is concentrated in states above the median difference, on average by 5.9 percentage‐points or 22% of the 2019 Medicaid coverage rates in those states. In contrast, there is no effect on Medicaid coverage in the states at or below that Median. Moreover, all effects on work outcomes in the above/at‐median states are small and not statistically significant but precisely estimated to rule out relatively small or moderate effects despite the smaller number of states in each stratified group. Specifically, the estimates rule out a decline of 2.2 percentage‐points in labor force participation, 1.5 percentage points in employment, and 0.9 percentage points in work hours, and 2.9 percentage points in full time employment, respectively. In the below‐median states, there is a statistically significant increase in work hours and full‐time employment which should be cautiously interpreted as likely reflecting other trends in this group considering the lack of effects on Medicaid coverage.

Lastly, we report the results from the heterogeneity analysis by demographic groups. First, Table A9 shows descriptive (unadjusted) postpartum Medicaid coverage rates by year for subgroups by age, race/ethnicity, and marital status. In any year, Medicaid rates are highest among younger (aged 19–25 years), non‐Hispanic Black, Hispanic, and single women, with all subgroups showing an increase in Medicaid coverage rates between 1 to 4 percentage‐points. Table A10 reports the difference‐in‐differences estimates of the FFCRA effects on Medicaid coverage and work outcomes across these demographic subgroups. The increase in Medicaid coverage is most pronounced among 19–25 years old (+5.8 percentage‐points;95% CI: 2.6 to 9.1), non‐Hispanic Black (+5.1 percentage‐points;95% CI: 0.3 to 9.8), and Hispanic (+4.5 percentage‐points; 95% CI: 1.9 to 7.1) women. The increase among single women is double that among married women but not statistically significant. For work outcomes, there is an increase in full to time employment (+2.1 percentage‐points; 95% CI: 0.2 to 3.9) among 19 to 25 years old women, an increase in labor force participation (+2.2 percentage to points; 95% CI: 0.2 to 4.2) and employment (+2.8 percentage‐points; 95% CI: 0.7 to 4.8) among Hispanic women, and an increase in employment (+2.2 percentage‐points; 95% CI: 0.6 to 3.8) among single women. Most other work outcome estimates for other subgroups are small and all are not statistically significant.

## 4 Discussion

The policy debate on adding work requirements for adult Medicaid beneficiaries has largely centered on concerns that beneficiaries may experience disincentives to work [22]. The extant literature offers mixed findings on previous Medicaid eligibility changes. Although work requirements have not applied to Medicaid coverage during pregnancy or postpartum, examining the effects of Medicaid coverage expansions during these periods on work outcomes is important for both understanding effects on low‐income families and informing the broader discussion on the premise of adding work requirements to adult Medicaid beneficiaries. We add to this literature by evaluating a recent policy, the FFCRA, which prohibited states from redetermining eligibility for Medicaid beneficiaries for 3 years between March 2020 and March 2023. For pregnant women, this policy extended postpartum coverage beyond 60 days (the pre‐FFCRA coverage window) for women at higher income levels than would be eligible for Medicaid after pregnancy; therefore, effectively expanding income eligibility for Medicaid beyond 60 days postpartum. Our study leverages differences in income eligibility between pregnancy and non‐pregnancy across states to examine the effects of this Medicaid income eligibility change on postpartum coverage and work outcomes.

We find an increase in Medicaid coverage following the FFCRA. The increase in Medicaid coverage is overall smaller than what has been reported in studies using data from the Pregnancy Risk Assessment Monitoring System (PRAMS) [12, 13, 14], a survey specific to women during postpartum but covers a shorter period after delivery (most interviews completed between 3 and 6 months after delivery) and has no questions on work outcomes. However, we find a larger increase in Medicaid coverage than the main sample estimate that is closer to the other study estimates when limiting the sample to states with a higher (above‐median) difference between pregnancy and non‐pregnancy eligibility.

In contrast, we find little evidence for effects on work outcomes. Changes in work outcomes are small, and most are not statistically significant. Importantly, the main sample results and those specific to states with higher differences between pregnancy and non‐pregnancy eligibility for which the increase in Medicaid coverage is pronounced are precise enough to rule out relatively small declines in employment and other evaluated work outcomes. Subgroup analyses show an increase in full‐time employment for 19–25‐year‐old women who had the largest increase in Medicaid coverage. There was also an increase in labor force participation and any employment for Hispanic women. Collectively, we view the findings as closer to those of studies finding no disemployment effects of the ACA Medicaid expansions on work outcomes [2, 4, 5] but somewhat different from those reporting Medicaid coverage to be associated with work decline, including one study of pregnancy eligibility expansions during the late 1980s and early 1990s [6, 8, 9]. However, certain factors may limit the generalizability of our findings on postpartum work outcomes to broader effects for other population groups such as parents of older children or childless adults. Specifically, access to or lack of childcare services and health insurance through a spouse or partner, and availability and preferences for maternity leave may be more salient in influencing decisions to return to work during postpartum than at another time. For example, the ability to return to work postpartum depends more on the availability of childcare services than when the child is of school age. Similarly, maternity leave availability and plans would also impact decisions on the timing of return to work, and with a lack of data on the month of interview after delivery, we are not able to separate effects over time. Such factors may attenuate the effects of continued or expanded Medicaid eligibility on postpartum work outcomes more than at another time and limit generalizability to other populations.

Our findings are useful for anticipating how the ARP extensions of postpartum coverage to 12 months affect work during this period. Moreover, the evidence from this study would be useful for any potential future policy proposals that may reconsider work requirements for parents with young children. Our findings can also contribute to the policy debate on work requirements for other adult Medicaid beneficiaries while considering potential differences in time use and work incentives between the two groups. We find no evidence for work decline across a range of demographic subgroups and so no evidence for effect heterogeneity across these demographic factors. Nonetheless, re‐examining the FFRCA effects on work outcomes of the general adult population of beneficiaries who also benefited from continued Medicaid eligibility in future research would be important to further understand those effects.

The FFCRA presents a mixture of two effects: (1) extending Medicaid eligibility at the same income level without re‐determining eligibility; and (2) allowing enrolled individuals to remain eligible if their income increases beyond the eligibility level after initial enrollment. Importantly for women during postpartum, the FFCRA represents a form of income eligibility expansion for women whose income during pregnancy and 60 days postpartum would have made them income ineligible after 60 days, as noted above. The study design captures this effect and the continuity of coverage effect for those women but removes the effect of continued coverage without needing to redetermine eligibility for women whose incomes would have kept them eligible post 60 days. Therefore, our findings might be most applicable to policies that expand income eligibility for specific periods with reducing eligibility redetermination. Different changes in Medicaid eligibility, such as increasing or reducing Medicaid income eligibility with or without modifying the frequency of eligibility determination, may affect work differently. Overall, our findings are within range of the collective evidence on effects of the ACA expansion for Medicaid eligibility for the general adult population that also points to no effects on work outcomes, which suggests somewhat similar effects on work between the two Medicaid eligibility changes in this case.

### 4.1 Limitations

Our study has strengths including a national and large sample, a difference‐in‐differences design, and examining a range of work outcomes that capture any work and work hours. There are also limitations. One limitation is that we are unable to determine the length of the postpartum period in the sample except that it ranges anywhere from a very recent delivery up to12 months after. Importantly, the estimates are meaningful as average effects for a sample of individuals interviewed at different times within 12 months postpartum. Also, to our knowledge, this would be the only national survey with a large enough sample to examine this research question of postpartum work outcomes.

Another potential caveat is that the FFCRA was enacted during the COVID‐19 pandemic which impacted work outcomes. Despite the initial sharp drop in employment, the labor market rebounded in 2021 and 2022, and the return to employment in this period may have conflated any negative effects on work outcomes from the continued eligibility for Medicaid. For such a potential confounding to affect the difference‐in‐differences estimates however, employment rebounding would have to systematically correlate with the state‐level differences between pregnancy and non‐pregnancy income eligibility which is not expected a priori. To address this concern, we estimate a model adding state unemployment rates to capture state‐specific labor market trends and find similar results. However, how effects compare to periods with economic downturns and national declines in labor demand would be an important future research question.

The is also underreporting of Medicaid coverage during the FFCRA period due to some beneficiaries being unaware of continued coverage [23]. However, we would not expect such underreporting to be correlated with the state‐level difference between pregnancy and non‐pregnancy eligibility on which rests the difference‐in‐differences design. In other words, a person who is covered by Medicaid should not differ in likelihood of underreporting based on the state of residence, and specifically the state‐level difference in income eligibility between pregnancy and non‐pregnancy. Therefore, underreporting should not bias the FFCRA effect estimates on coverage and work outcomes as estimated.

Finally, generalizability to other contexts is subject to whether effects differ by the nature of the Medicaid eligibility change, such as income eligibility expansions versus reducing the frequency of eligibility determination, and the affected population considering potential differences in work incentives and time use between women within 12 months from delivery and the general population of adults as noted above. Continuing to examine the FFCRA effects and other Medicaid eligibility expansions for other populations is important to further understand how Medicaid coverage affects work incentives in various contexts.

## 5 Conclusion

This study adds to the evidence on Medicaid coverage effects on work outcomes by examining a recent policy, the FFCRA, which effectively expanded income eligibility for postpartum Medicaid coverage beyond 60 days by prohibiting re‐eligibility determination. There is clear evidence that the FFCRA increased Medicaid coverage through 2022, the last year of the study, but little evidence of effects on work outcomes. Specifically, there is no evidence of a decline in work with precise estimates to rule out relatively small declines across a range of outcomes. These findings indicate that the subsequent state‐level extensions of postpartum Medicaid coverage to 12 months are unlikely to disincentivize work for women benefiting from this coverage.

## Disclosure

The authors have nothing to report.

## Conflicts of Interest

The authors declare no conflicts of interest.

## Supporting information


**Data S1:** Supporting Information.

## Data Availability

The data that support the findings of this study are available in IPUMS at https://usa.ipums.org/usa/. These data were derived from the following resources available in the public domain: ‐ IPUMS, https://usa.ipums.org/usa/.
