# Supplementary material for: Effects of Medicaid Coverage on Work: Evidence From Extending Postpartum Medicaid Coverage
Source: Health Serv Res. 2025 Oct 9;61(1):e70055. doi: 10.1111/1475-6773.70055 (PMC12857479; doi:10.1111/1475-6773.70055)
Supplement: Supplementary file 1 — Data S1: Supporting Information. [file HESR-61-0-s001.docx]

**Online Appendix**

[Appendix Table 1. Medicaid Expansion Status and Medicaid Income Eligibility for Pregnant Women and Parents in 2019 among 42 States Included in the Main Analytical Sample 2](#_Toc207876377)

[Appendix Table 2. Descriptive Statistics for the Full Sample 4](#_Toc207876378)

[Appendix Table 3. Effects of FFCRA Medicaid Continuous Coverage Provision on Medicaid Coverage and Work Outcomes, Women Aged 19-49 Years with a Child Born within the Past 12 Months, 2016-2019 and 2021-2022 ACS (2020 Data Excluded) 5](#_Toc207876379)

[Appendix Table 4. Effects of FFCRA Medicaid Continuous Coverage Provision on Any Insurance Coverage and Private Coverage by Type, Women Aged 19-49 Years with a Child Born within the Past 12 Months for the Full Sample, 2016-2022 ACS 6](#_Toc207876380)

[Appendix Table 5. Effects of FFCRA Medicaid Continuous Coverage Provision on Any Insurance Coverage and Private Coverage by Type, Women Aged 19-49 Years with a Child Born within the Past 12 Months, 2016-2019 and 2021-2022 ACS (2020 Data Excluded) 7](#_Toc207876381)

[Appendix Table 6. Effects of FFCRA Medicaid Continuous Coverage Provision on Medicaid Coverage and Work Outcomes, Effects Scaled by the 75^th^ and 90^th^ Percentiles of the Income Eligibility Difference between Pregnancy and non-Pregnancy, Women Aged 19-49 Years with a Child Born within the Past 12 Months for the Full Sample, 2016-2022 ACS 8](#_Toc207876382)

[Appendix Table 7. Effects of FFCRA Medicaid Continuous Coverage Provision on Medicaid Coverage and Work Outcomes, Women Aged 19-49 Years with a Child Born within the Past 12 Months for the Full Sample with Controlling for State Unemployment Rate, 2016-2022 ACS 9](#_Toc207876383)

[Appendix Table 8. Effects of FFCRA Medicaid Continuous Coverage Provision on Medicaid Coverage and Work Outcomes by the Median Difference between Pregnancy and Parent eligibility, Women Aged 19-49 Years with a Child Born within the Past 12 Months , 2016-2022 ACS 10](#_Toc207876384)

[Appendix Table 9. Descriptive Statistics Showing Postpartum Women’s Medicaid Rates (%) by Subgroups by Year for Full Sample 11](#_Toc207876385)

[Appendix Table 10. Sub-group Analysis of the FFCRA Medicaid Continuous Coverage Provision on Medicaid Coverage and Work Outcomes, Women Aged 19-49 Years with a Child Born within the Past 12 Months, 2016-2022 ACS 12](#_Toc207876386)

| Appendix Table 1. Medicaid Expansion Status and Medicaid Income Eligibility for Pregnant Women and Parents in 2019 among 42 States Included in the Main Analytical Sample | | | | | |
| --- | --- | --- | --- | --- | --- |
| State | ACA Medicaid Expansion Date  (As of December 2022) | 2019 Medicaid Income Eligibility for Pregnant Women (% FPL) | 2019 Medicaid Income Eligibility for Pregnant Parents (% FPL) | Differences in 2019 Medicaid Eligibility between Pregnant Women and Parents (% FPL) | States below/at median differences between parent and pregnancy eligibility levels |
| Alabama | No Expansion | 146 | 18 | 128 | No |
| Alaska | 09/01/2015 | 205 | 138 | 67 | Yes |
| Arizona | 01/01/2014 | 161 | 138 | 23 | Yes |
| Arkansas | 01/01/2014 | 214 | 138 | 76 | Yes |
| California | 01/01/2014 | 213 | 138 | 75 | Yes |
| Colorado | 01/01/2014 | 200 | 138 | 62 | Yes |
| Connecticut | 01/01/2014 | 263 | 155 | 108 | No |
| Delaware | 01/01/2014 | 217 | 138 | 79 | Yes |
| District of Columbia | 01/01/2014 | 324 | 221 | 103 | No |
| Florida | No Expansion | 196 | 32 | 164 | No |
| Georgia | No Expansion | 225 | 35 | 190 | No |
| Hawaii | 01/01/2014 | 196 | 138 | 58 | Yes |
| Illinois | 01/01/2014 | 213 | 138 | 75 | Yes |
| Indiana | 02/01/2015 | 218 | 139 | 79 | Yes |
| Iowa | 01/01/2014 | 380 | 138 | 242 | No |
| Kansas | No Expansion | 171 | 38 | 133 | No |
| Kentucky | 01/01/2014 | 200 | 138 | 62 | Yes |
| Maryland | 01/01/2014 | 264 | 138 | 126 | No |
| Massachusetts | 01/01/2014 | 205 | 138 | 67 | Yes |
| Michigan | 04/01/2014 | 200 | 138 | 62 | Yes |
| Minnesota | 01/01/2014 | 283 | 138 | 145 | No |
| Mississippi | No Expansion | 199 | 26 | 173 | No |
| Nevada | 01/01/2014 | 165 | 138 | 27 | Yes |
| New Hampshire | 08/15/2014 | 201 | 138 | 63 | Yes |
| New Jersey | 01/01/2014 | 199 | 138 | 61 | Yes |
| New Mexico | 01/01/2014 | 255 | 138 | 117 | No |
| New York | 01/01/2014 | 223 | 138 | 85 | No |
| North Carolina | No Expansion | 201 | 42 | 159 | No |
| North Dakota | 01/01/2014 | 152 | 138 | 14 | Yes |
| Ohio | 01/01/2014 | 205 | 138 | 67 | Yes |
| Oregon | 01/01/2014 | 190 | 138 | 52 | Yes |
| Pennsylvania | 01/01/2015 | 220 | 138 | 82 | Yes |
| Rhode Island | 01/01/2014 | 195 | 138 | 57 | Yes |
| South Carolina | No Expansion | 199 | 67 | 132 | No |
| South Dakota | No Expansion | 138 | 49 | 89 | No |
| Tennessee | No Expansion | 200 | 95 | 105 | No |
| Texas | No Expansion | 203 | 17 | 186 | No |
| Vermont | 01/01/2014 | 213 | 138 | 75 | Yes |
| Washington | 01/01/2014 | 198 | 138 | 60 | Yes |
| West Virginia | 01/01/2014 | 163 | 138 | 25 | Yes |
| Wisconsin | No Expansion | 306 | 100 | 206 | No |
| Wyoming | No Expansion | 159 | 54 | 105 | No |
| The status of Medicaid expansion and income eligibility for Medicaid are obtained from *Annual Updates on Eligibility Rules, Enrollment and Renewal Procedures, and Cost-Sharing Practices in Medicaid and CHIP* from Kaiser Family Foundation. Median differences in parent and pregnancy eligibility levels (79% FPL).  Abbreviations: ACA=Affordable Care Act; FPL=Federal Poverty Level; CHIP=Child Health Insurance Program. | | | | | |

| Appendix Table 2. Descriptive Statistics for the Full Sample | | |
| --- | --- | --- |
|  | 2016-2019 | 2020-2022 |
| **Postpartum Coverage Outcomes** |  |  |
| Medicaid Coverage (%) | 29.75 | 30.16 |
| Any Coverage (%) | 88.53 | 89.96 |
| Private Coverage (%) | 59.91 | 61.94 |
| Employer-Sponsored Health Coverage (%) | 54.09 | 55.91 |
| Privately Purchased Coverage (%) | 7.39 | 8.19 |
|  |  |  |
| **Labor Outcomes** |  |  |
| Labor Force participation (%) | 65.16 | 67.07 |
| Employment status (%) | 60.55 | 62.24 |
| Working Hours per Week | 25.07 | 25.97 |
| Fulltime employment (%) | 55.47 | 58.14 |
|  |  |  |
| **Covariates** |  |  |
| Race/Ethnicity (%) |  |  |
| Non-Hispanic Whites | 51.93 | 50.42 |
| Non-Hispanic Blacks | 14.10 | 13.30 |
| Non-Hispanic Other Races | 10.60 | 12.15 |
| Hispanics | 23.36 | 24.13 |
|  |  |  |
| Maternal Age | 30.46 | 31.17 |
|  |  |  |
| Maternal Education (%) |  |  |
| Less than High School | 10.55 | 9.05 |
| High School | 23.37 | 22.58 |
| Some Colleges | 30.84 | 28.85 |
| College and above | 35.25 | 39.52 |
|  |  |  |
| Married (%) | 65.22 | 68.21 |
|  |  |  |
| Number of Children | 1.83 | 1.83 |
|  |  |  |
| Number of Adults | 2.28 | 2.26 |
|  |  |  |
| US Citizenship Status | 78.01 | 78.71 |
|  |  |  |
| **Number of Observations** | 121408 | 83696 |
| Notes: The descriptive statistics are derived from the analytical sample among women aged 19-44 years old who gave a birth within the past 12 months. All statistics are weighted using the sampling weights. Variables without a “%” label are all continuous variables. | | |

| Appendix Table 3. Effects of FFCRA Medicaid Continuous Coverage Provision on Medicaid Coverage and Work Outcomes, Women Aged 19-49 Years with a Child Born within the Past 12 Months, 2016-2019 and 2021-2022 ACS (2020 Data Excluded) | | | | | |
| --- | --- | --- | --- | --- | --- |
|  | Medicaid Coverage | Labor Force Participation | Employment Status | Working Hours per Week^+^ | Fulltime Employment Status^+^ |
| Effect Estimate  (2021-2022) | 3.1** | 0.10 | 0.5 | 0.09 | 0.6 |
| SE | (1.1) | (0.5) | (0.4) | (0.2) | (0.5) |
| 95% CI | [1.0,5.2] | [-1.0,1.2] | [-0.3,1.3] | [-0.3,0.5] | [-0.5,1.7] |
| N | 182195 | 182195 | 182195 | 182195 | 182195 |
| Outcome Mean  (2016-2019) | 29.7 | 65.2 | 60.6 | 25.1 | 55.5 |
| Notes: This table reports the effects of FFCRA Medicaid continuous coverage provision on postpartum health insurance and work outcomes. Estimates are from a difference-in-differences regression leveraging differences across states in income eligibility between pregnancy and non-pregnancy and overtime (2021-2022 versus 2016-2019). Effects are scaled to the average difference in this income eligibility in 2019 (97% FPL). The model adjusts for maternal age, education, race/ethnicity, marital status, number of adults and children in household, unemployment rates, US citizenship status, state and year fixed effects. All regressions are estimated using the least squares with the sampling probability weights provided in the ACS data.  * p<0.05, **p<0.01, ***p<0.001; ^+^ Outcomes include zeros for unemployed individuals and those not in the labor force. Abbreviations: FFCRA=Families First Coronavirus Response Act (FFCRA); ACS=American Community Survey; SE=Standard Errors; CI=Confidence Interval; FPL=Federal Poverty Level | | | | | |

| Appendix Table 4. Effects of FFCRA Medicaid Continuous Coverage Provision on Any Insurance Coverage and Private Coverage by Type, Women Aged 19-49 Years with a Child Born within the Past 12 Months for the Full Sample, 2016-2022 ACS | | | | |
| --- | --- | --- | --- | --- |
|  | Any Insurance Coverage | Any Private Coverage | Employer-Sponsored  Health Coverage | Privately Purchased Coverage |
| Effect Estimate (2020-2022) | 2.3*** | -0.3 | -0.2 | 0.001 |
| SE | (0.5) | (0.7) | (0.7) | (0.4) |
| 95% CI | [1.2,3.3] | [-1.7,1.1] | [-1.6,1.1] | [-0.8,0.8] |
| N | 205104 | 205104 | 205104 | 205104 |
| Outcome Mean (2016-2019) | 88.5 | 59.9 | 54.1 | 7.4 |
| Notes: This table reports the effects of FFCRA Medicaid continuous coverage provision on postpartum health insurance and work outcomes. Estimates are from a difference-in-differences regression leveraging differences across states in income eligibility between pregnancy and non-pregnancy and over time (2020-2022 versus 2016-2019). Effects are scaled to the average difference in this income eligibility in 2019 (97% FPL). The model adjusts for maternal age, education, race/ethnicity, marital status, number of adults and children in household, US citizenship status, state and year fixed effects. All regressions are estimated using the least squares with the sampling probability weights provided in the ACS data.  * p<0.05, **p<0.01, ***p<0.001; ^+^ Outcomes include zeros for unemployed individuals and those not in the labor force.  Abbreviations: FFCRA=Families First Coronavirus Response Act (FFCRA); ACS=American Community Survey; SE=Standard Errors; CI=Confidence Interval; FPL=Federal Poverty Level | | | | |

| Appendix Table 5. Effects of FFCRA Medicaid Continuous Coverage Provision on Any Insurance Coverage and Private Coverage by Type, Women Aged 19-49 Years with a Child Born within the Past 12 Months, 2016-2019 and 2021-2022 ACS (2020 Data Excluded) | | | | |
| --- | --- | --- | --- | --- |
|  | Any Insurance Coverage | Any Private Coverage | Employer-Sponsored  Health Coverage | Privately Purchased Coverage |
| Effect Estimate (2021-2022) | 2.7*** | 0.1 | 0.1 | 0.3 |
| SE | (0.6) | (0.8) | (0.7) | (0.5) |
| 95% CI | [1.5,3.9] | [-1.6,1.8] | [-1.3,1.6] | [-0.8,1.3] |
| N | 182195 | 182195 | 182195 | 182195 |
| Outcome Mean (2016-2019) | 88.5 | 59.9 | 54.1 | 7.4 |
| Notes: This table reports the effects of FFCRA Medicaid continuous coverage provision on postpartum health insurance and work outcomes. Estimates are from a difference-in-differences regression leveraging differences across states in income eligibility between pregnancy and non-pregnancy and overtime (2021-2022 versus 2016-2019). Effects are scaled to the average difference in this income eligibility in 2019 (97% FPL). The model adjusts for maternal age, education, race/ethnicity, marital status, number of adults and children in household, US citizenship status, state and year fixed effects. All regressions are estimated using the least squares with the sampling probability weights provided in the ACS data.  * p<0.05, **p<0.01, ***p<0.001; ^+^ Outcomes include zeros for unemployed individuals and those not in the labor force.  Abbreviations: FFCRA=Families First Coronavirus Response Act (FFCRA); ACS=American Community Survey; SE=Standard Errors; CI=Confidence Interval; FPL=Federal Poverty Level | | | | |

| Appendix Table 6. Effects of FFCRA Medicaid Continuous Coverage Provision on Medicaid Coverage and Work Outcomes, Effects Scaled by the 75^th^ and 90^th^ Percentiles of the Income Eligibility Difference between Pregnancy and non-Pregnancy, Women Aged 19-49 Years with a Child Born within the Past 12 Months for the Full Sample, 2016-2022 ACS | | | | | |
| --- | --- | --- | --- | --- | --- |
|  | Medicaid Coverage | Labor Force Participation | Employment Status | Working Hours per Week^+^ | Fulltime Employment Status^+^ |
| Effect Scaled by 75 Percentile |  |  |  |  |  |
| Effect Estimate (2020-2022) | 3.7** | 0.2 | 0.6 | 0.08 | 0.3 |
| SE | (1.3) | (0.7) | (0.6) | (0.3) | (0.9) |
| 95% CI | [1.1,6.4] | [-1.2,1.7] | [-0.5,1.7] | [-0.5,0.7] | [-1.5,2.1] |
|  |  |  |  |  |  |
| Effect Scaled by 90 Percentile |  |  |  |  |  |
| Effect Estimate (2020-2022) | 5.0** | 0.3 | 0.8 | 0.1 | 0.4 |
| SE | (1.8) | (1.0) | (0.8) | (0.4) | (1.2) |
| 95% CI | [1.4,8.6] | [-1.7,2.3] | [-0.7,2.3] | [-0.7,0.9] | [-2.0,2.8] |
|  |  |  |  |  |  |
| N | 205104 | 205104 | 205104 | 205104 | 205104 |
| Outcome Mean (2016-2019) | 29.7 | 65.2 | 60.6 | 25.1 | 55.5 |
| Notes: This table reports the effects of FFCRA Medicaid continuous coverage provision on postpartum health insurance and work outcomes. Estimates are from a difference-in-differences regression leveraging differences across states in income eligibility between pregnancy and non-pregnancy and over time (2020-2022 versus 2016-2019). Effects are scaled by the 75 percentile (128% FPL) and 90 percentile (173% FPL) of the difference in income eligibility in 2019 . The model adjusts for maternal age, education, race/ethnicity, marital status, number of adults and children in household, unemployment rates, US citizenship status, state and year fixed effects. All regressions are estimated using the least squares with the sampling probability weights provided in the ACS data.  * p<0.05, **p<0.01, ***p<0.001; ^+^ Outcomes include zeros for unemployed individuals and those not in the labor force.  Abbreviations: FFCRA=Families First Coronavirus Response Act (FFCRA); ACS=American Community Survey; SE=Standard Errors; CI=Confidence Interval; FPL=Federal Poverty Level | | | | | |

| Appendix Table 7. Effects of FFCRA Medicaid Continuous Coverage Provision on Medicaid Coverage and Work Outcomes, Women Aged 19-49 Years with a Child Born within the Past 12 Months for the Full Sample with Controlling for State Unemployment Rate, 2016-2022 ACS | | | | | |
| --- | --- | --- | --- | --- | --- |
|  | Medicaid Coverage | Labor Force Participation | Employment Status | Working Hours per Week^+^ | Fulltime Employment Status^+^ |
| Effect Estimate (2020-2022) | 2.8** | 0.2 | 0.4 | 0.06 | 0.2 |
| SE | (1.0) | (0.5) | (0.4) | (0.2) | (0.7) |
| 95% CI | [0.8,4.8] | [-0.9,1.3] | [-0.4,1.3] | [-0.4,0.5] | [-1.1,1.6] |
| N | 205104 | 205104 | 205104 | 205104 | 205104 |
| Outcome Mean (2016-2019) | 29.7 | 65.2 | 60.6 | 25.1 | 55.5 |
| Notes: This table reports the effects of FFCRA Medicaid continuous coverage provision on postpartum health insurance and work outcomes. Estimates are from a difference-in-differences regression leveraging differences across states in income eligibility between pregnancy and non-pregnancy and over time (2020-2022 versus 2016-2019). Effects are scaled to the average difference in this income eligibility in 2019 (97% FPL). The model adjusts for maternal age, education, race/ethnicity, marital status, number of adults in household, number of children in household, state unemployment rates, US citizenship status, state and year fixed effects. All regressions are estimated using the least squares with the sampling probability weights provided in the ACS data.  * p<0.05, **p<0.01, ***p<0.001; ^+^ Outcomes include zeros for unemployed individuals and those not in the labor force.  Abbreviations: FFCRA=Families First Coronavirus Response Act (FFCRA); ACS=American Community Survey; SE=Standard Errors; CI=Confidence Interval; FPL=Federal Poverty Level | | | | | |

| Appendix Table 8. Effects of FFCRA Medicaid Continuous Coverage Provision on Medicaid Coverage and Work Outcomes by the Median Difference between Pregnancy and Parent eligibility, Women Aged 19-49 Years with a Child Born within the Past 12 Months , 2016-2022 ACS | | | | | |
| --- | --- | --- | --- | --- | --- |
|  | Medicaid Coverage | Labor Force Participation | Employment Status | Working Hours per Week^+^ | Fulltime Employment Status^+^ |
| States below or at Median^1^ |  |  |  |  |  |
| Effect Estimate (2020-2022) | -0.2 | 0.3 | 0.2 | 1.2* | 4.2** |
| SE | (1.8) | (0.8) | (0.9) | (0.4) | (1.3) |
| 95% CI | [-4.1,3.6] | [-1.3,1.9] | [-1.7,2.1] | [0.3, 2.0] | [1.6,6.9] |
| N | 98517 | 98517 | 98517 | 98517 | 98517 |
| Outcome Mean (2016-2019) | 33.2 | 64.6 | 60.2 | 24.7 | 54.3 |
| States Above Median^1^ |  |  |  |  |  |
| Effect Estimate (2020-2022) | 5.9* | 0.3 | 0.3 | 0.2 | 0.07 |
| SE | (2.4) | (1.2) | (0.9) | (0.5) | (1.4) |
| 95% CI | [0.9,10.9] | [-2.2,2.8] | [-1.5,2.1] | [-0.9,1.2] | [-2.9,3.0] |
| N | 106587 | 106587 | 106587 | 106587 | 106587 |
| Outcome Mean (2016-2019) | 26.7 | 65.6 | 60.9 | 25.4 | 56.5 |
|  |  |  |  |  |  |
| Notes: This table reports the effects of FFCRA Medicaid continuous coverage provision on postpartum health insurance and work outcomes. Estimates are from a difference-in-differences regression leveraging differences across states in income eligibility between pregnancy and non-pregnancy and over time (2020-2022 versus 2016-2019). Effects are scaled to the average difference in this income eligibility in 2019 by state in specific group based on the median difference. The model adjusts for maternal age, education, race/ethnicity, marital status, number of adults in household, number of children in household, US citizenship status, state and year fixed effects. All regressions are estimated using the least squares with the sampling probability weights provided in the ACS data.  ^1^ Estimates are scaled by the average difference between pregnancy and non-pregnancy eligibility specific to each group (79% of the FPL in the above/at-median group and 58% of the FPL in the below-median group). Below/at median: Alaska, Arizona, Arkansas, California, Colorado, Delaware, Hawaii, Illinois, Indiana, Kentucky, Massachusetts, Michigan, Nevada, New Hampshire, New Jersey, North Dakota, Ohio, Oregon, Pennsylvania, Rhode Island, Vermont, Washington, West Virginia. Above the Median: Alabama, Connecticut, District of Columbia, Florida, Georgia, Iowa, Kansas, Maryland, Minnesota, Mississippi, New Mexico, New York, North Carolina, South Carolina, South Dakota, Tennessee, Texas, Wisconsin, Wyoming.  * p<0.05, **p<0.01, ***p<0.001; ^+^ Outcomes include zeros for unemployed individuals and those not in the labor force.  Abbreviations: FFCRA=Families First Coronavirus Response Act (FFCRA); ACS=American Community Survey; SE=Standard Errors; CI=Confidence Interval; FPL=Federal Poverty Level | | | | | |

| Appendix Table 9. Descriptive Statistics Showing Postpartum Women’s Medicaid Rates (%) by Subgroups by Year for Full Sample | | | | | | | | |
| --- | --- | --- | --- | --- | --- | --- | --- | --- |
|  | 2016 | 2017 | 2018 | 2019 | 2020 | 2021 | 2022 | ***Difference between 2022 and 2019*** |
| **By Age** |  |  |  |  |  |  |  |  |
| 19-25 | 49.92 | 48.98 | 47.25 | 47.88 | 48.15 | 51.45 | 51.30 | 3.42 |
| 26-34 | 26.85 | 26.79 | 26.67 | 25.46 | 26.69 | 29.13 | 27.93 | 2.47 |
| 35-49 | 21.35 | 20.33 | 18.22 | 18.42 | 17.83 | 21.59 | 21.95 | 3.53 |
|  |  |  |  |  |  |  |  |  |
| **By Race/Ethnicity** |  |  |  |  |  |  |  |  |
| Non-Hispanic Whites | 25.03 | 23.94 | 22.66 | 22.50 | 22.34 | 25.00 | 23.51 | 1.01 |
| Non-Hispanic Blacks | 46.95 | 46.36 | 45.49 | 44.48 | 45.36 | 48.79 | 48.47 | 3.99 |
| Non-Hispanic Others | 27.59 | 25.01 | 24.97 | 23.25 | 25.03 | 28.14 | 27.00 | 3.75 |
| Hispanics | 37.74 | 37.12 | 34.83 | 33.77 | 33.90 | 36.87 | 36.91 | 3.14 |
|  |  |  |  |  |  |  |  |  |
| **By Marital Status** |  |  |  |  |  |  |  |  |
| Married | 18.99 | 17.92 | 16.80 | 16.31 | 17.68 | 19.47 | 19.41 | 3.10 |
| Single | 53.24 | 53.86 | 51.84 | 51.79 | 52.89 | 55.65 | 54.55 | 2.76 |
| Notes: The descriptive statistics are derived from the analytical sample among women aged 19-49 years old who gave a birth within the past 12 months. All statistics are weighted using the sampling weights. | | | | | | | | |

| Appendix Table 10. Sub-group Analysis of the FFCRA Medicaid Continuous Coverage Provision on Medicaid Coverage and Work Outcomes, Women Aged 19-49 Years with a Child Born within the Past 12 Months, 2016-2022 ACS | | | | | | | |
| --- | --- | --- | --- | --- | --- | --- | --- |
|  | N | Medicaid Coverage | Labor Force Participation | Employment Status | Working Hours per Week^+^ | Fulltime Employment Status^+^ |  |
| By Age |  |  |  |  |  |  |  |
| 19-25 | 39164 | 5.8*** | 1.1 | 1.4 | 0.4 | 2.1* |  |
|  |  | (1.6) | (1.0) | (1.0) | (0.3) | (0.9) |  |
|  |  | [2.6,9.1] | [-0.9,3.0] | [-0.5,3.4] | [-0.3,1.1] | [0.2,3.9] |  |
| 26-34 | 108917 | 2.2* | -0.4 | 0.2 | -0.09 | -0.6 |  |
|  |  | (1.0) | (0.7) | (0.6) | (0.2) | (0.7) |  |
|  |  | [0.2,4.2] | [-1.9,1.0] | [-0.9,1.3] | [-0.6,0.4] | [-2.0,0.8] |  |
| 35-49 | 57023 | 0.4 | 0.7 | 1.5 | 0.1 | 0.6 |  |
|  |  | (0.7) | (0.7) | (0.7) | (0.4) | (1.1) |  |
|  |  | [-1.1,1.9] | [-0.7,2.2] | [-0.02,3.0] | [-0.7,0.9] | [-1.6,2.8] |  |
| By Race/Ethnicity |  |  |  |  |  |  |  |
| Non-Hispanic Whites | 119483 | 1.4* | 0.03 | 0.9 | -0.2 | 0.10 |  |
|  |  | (0.7) | (0.8) | (0.8) | (0.4) | (1.0) |  |
|  |  | [0.01,2.8] | [-1.5,1.6] | [-0.8,2.5] | [-0.9,0.5] | [-2.0,2.2] |  |
| Non-Hispanic Blacks | 19103 | 5.1* | 0.006 | 0.3 | 1.0 | 1.8 |  |
|  |  | (2.3) | (1.3) | (1.7) | (0.5) | (1.5) |  |
|  |  | [0.3,9.8] | [-2.7,2.7] | [-3.2,3.7] | [-0.03,2.1] | [-1.1,4.7] |  |
| Non-Hispanic Others | 24342 | 2.5 | -1.8 | -1.9 | -0.6 | -2.7 |  |
|  |  | (1.3) | (1.2) | (1.2) | (0.5) | (1.5) |  |
|  |  | [-0.005,5.1] | [-4.2,0.5] | [-4.4,0.6] | [-1.5,0.3] | [-5.8,0.3] |  |
| Hispanics | 42176 | 4.5** | 2.2* | 2.8** | 0.5 | 1.2 |  |
|  |  | (1.3) | (1.0) | (1.0) | (0.4) | (0.8) |  |
|  |  | [1.9,7.1] | [0.2,4.2] | [0.7,4.8] | [-0.3,1.3] | [-0.5,2.9] |  |
| By Marital Status |  |  |  |  |  |  |  |
| Married | 146765 | 1.8** | -0.5 | 0.08 | -0.2 | -0.5 |  |
|  |  | (0.6) | (0.4) | (0.4) | (0.3) | (0.8) |  |
|  |  | [0.6,3.0] | [-1.4,0.3] | [-0.8,1.0] | [-0.8,0.3] | [-2.1,1.1] |  |
| Single | 58339 | 4.0 | 1.5 | 2.2** | 0.7 | 1.6 |  |
|  |  | (2.0) | (1.3) | (0.8) | (0.3) | (0.9) |  |
|  |  | [-0.08,8.1] | [-1.1,4.1] | [0.6,3.8] | [-0.05,1.4] | [-0.2,3.5] |  |
| Notes: Standard errors are in parentheses and clustered by state. 95% confidence intervals are reported in brackets. This table reports the effects of FFCRA Medicaid continuous coverage provision on postpartum health insurance and work outcomes. Estimates are from a difference-in-differences regression leveraging differences across states in income eligibility between pregnancy and non-pregnancy and over time (2020-2022 versus 2016-2019). Effects are scaled to the average difference in this income eligibility in 2019 (97% FPL). The model adjusts for maternal age, education, race/ethnicity, marital status, number of adults in household, number of children in household, US citizenship status, state and year fixed effects. All regressions are estimated using the least squares with the sampling probability weights provided in the ACS data.  * p<0.05, **p<0.01, ***p<0.001; ^+^ Outcomes include zeros for unemployed individuals and those not in the labor force.  Abbreviations: FFCRA=Families First Coronavirus Response Act (FFCRA);ACS=American Community Survey; FPL=Federal Poverty Level | | | | | | | |
